# The Blood Lead Levels of Children and the Loss of Ca^2+^ from Neurons Owing to Lead

**DOI:** 10.3390/ijerph182212051

**Published:** 2021-11-17

**Authors:** Yifei Duan, Hua Shi, Yongmei Jiang

**Affiliations:** 1 Department of Laboratory Medicine, West China Second University Hospital, Sichuan University, Chengdu 610041, China; dyf2021@163.com (Y.D.); hs_scu@126.com (H.S.); 2 Key Laboratory of Birth Defects and Related Diseases of Women and Children, Ministry of Education, Sichuan University, Chengdu 610041, China

**Keywords:** blood lead levels, treatment status, calcium supplement, Ca^2+^ imaging

## Abstract

In order to understand current blood lead levels (BLLs), we investigated the BLLs of children in Sichuan Province from 2011 to 2020. We then monitored the treatment effects of calcium in children with high BLLs to assess their treatment status. Finally, we explored the effects of lead on Ca^2+^ through in-situ experiments. Whole blood samples were used for BLL tests. The BLLs of 76,362 children aged 0–7 years were measured using atomic absorption spectrometry. The median BLL was 35 μg/L (interquartile range: 28–47). The BLLs were significantly higher in boys than in girls (*p* < 0.001). The BLLs generally decreased annually and increased with age. The overall prevalence of BLLs ≥ 100 μg/L was 1.20%. The children with high BLLs received subsequent check-ups, and the median time required for effective treatment was 18 months. We observed that lead exposure led to a gradual and persistent loss of Ca^2+^ levels in neurons of mice brain slices, and the effect did not subside immediately even after the lead was removed. China has made rapid progress in pediatric healthcare, but the treatment status remains unsatisfactory. Because lead causes an irreversible loss of Ca^2+^, there is an urgent need to develop new standardized treatments to reduce the treatment duration.

## 1. Introduction

Lead is a non-biodegradable, malleable, noncorrosive heavy metal. As it has favorable properties for many man-made applications, it has been used for centuries. Lead exists in our daily lives in the composition of various pigments, glazes, paints, and plastic products. It can also be discharged into the atmosphere during the mining or processing of lead or lead-bearing ores as well as amidst the manufacturing of lead products and batteries. Unfortunately, lead may enter the human body through air, water, and food, and it can accumulate in the organs and further affect their functions [1,2]. Young children are more likely to suffer the impacts of lead exposure owing to their hand-to-mouth habits, weak immune system, incomplete blood–brain barrier, and higher absorption and lower discharge capacity [3,4]. Even though the incidence of lead poisoning in children has recently decreased, the hazards of lead exposure, such as deficits in neural plasticity, learning, and memory, should not be underestimated [5,6]. Lead-induced damage in the prefrontal cerebral cortex, hippocampus, and cerebellum can lead to a variety of neurologic disorders. High BLLs and lead poisoning remain as serious concerns, and effective methods to avoid lead exposure are yet to be developed. Early screening, standardized treatment, and regular follow-ups may prove useful in children at high risk of lead exposure.

Two documents titled “The Children Lead Acidosis and Lead Poisoning Prevention Guide” and “Children’s Blood Lead and Lead Poisoning Classification Principles” [7] were issued by the Ministry of Health in China as guidelines for the prevention, diagnosis, and treatment of lead poisoning. Lead poisoning has been classified into three grades, namely severe (BLLs ≥ 450 μg/L), moderate (250 ≤ BLLs ≤ 449 μg/L), and mild (200 ≤ BLLs ≤ 249 μg/L) lead poisoning, and BLLs between 100 and 199 μg/L for two consecutive tests can be considered as high blood lead. As per the abovementioned documents, treatment for children with 100 ≤ BLLs ≤ 249 μg/L includes keeping the children away from lead pollution sources, paying attention to hygiene, and nutrition intervention; for children with BLLs ≥ 250 μg/L, additional chelation therapy is also needed. The documents detail chelation therapy for children with moderate and severe lead poisoning; however, they do not specify standardized nutritional interventions for children with high blood lead and mild lead poisoning. An oral calcium supplement is often used to address high blood lead and mild lead poisoning in clinical practice. For children with BLLs between 100 and 250 μg/L, taking an oral calcium supplement for more than half a year may slowly reduce the BLL. Long-term exposure to high BLLs can greatly affect the growth and development of young children, especially their neural systems. Considering the great hazard of lead toxicity, the Centers for Disease Control and Prevention, USA, ascertained the BLL that should prompt public health actions as 100 μg/L in 1991, and they subsequently adjusted it to 50 μg/L, highlighting, however, that no levels of lead may be considered “safe” for children. Studies have proved that even BLLs that are lower than the cut-off may affect the development of the neural system of a child [8]. Therefore, the grading and treatment strategy for lead poisoning and high blood lead, respectively, in China may need to be modified and improved.

Moreover, the mechanisms by which lead disrupts the brain and its behavior are complex and remain poorly understood and thus are active research topics. The antagonistic behavior between lead and calcium is of particular significance. Lead binds to the sites at which calcium typically acts and enters the cells through calcium channels; thus, it substitutes for calcium in the inhibition and/or activation of calcium-dependent processes [9]. Lead binds to voltage-gated calcium channels and reduces the influx of calcium, thereby reducing the release of neurotransmitters [10,11,12]. The disruption of normal neurotransmitter release may result in a variety of consequences for the brain and its behavior depending on the specific neurotransmitter and its location in the brain. To observe the interaction between lead and Ca^2+^ in the cerebral cortex and to discern the effects of lead on the influx of calcium, the neurons were observed using dynamic Ca^2+^ imaging under lead exposure. Ca^2+^ imaging provides a wide range of applications in neuroscience because it can measure neuronal dynamics and network activity. It monitors network-level changes in neurons. Ca^2+^ imaging typically reveals some spontaneous neuronal firing by detecting dramatic changes in the level of Ca^2+^ [13]. Therefore, Ca^2+^ imaging not only reflects the change of Ca^2+^-level in neurons caused by lead but also reflects the effect of lead on neuron function.

Lead exposure in children in Sichuan Province has not been reported recently; therefore, the current study aims to update the BLL data for children aged 0–7 years in this area based on the past decade and trace the efficiency of treatment in children with high BLLs. Moreover, the antagonistic effects of lead against Ca^2+^ in neurons under lead exposure are observed using Ca^2+^ imaging.

## 2. Materials and Methods

### 2.1. Ethics Statement

Ethics approval was obtained from the Medical Ethics Committee of West China Second University Hospital of Sichuan University (protocol 2021176). All experiments using mice were approved by the Animal Research Committee at the West China Second University Hospital of Sichuan University (protocol 2021048A).

### 2.2. Participants

The study recruited 76,362 children who received regular health check-ups at the outpatient clinic of West China Second University Hospital of Sichuan University between 2011 and 2020. All the children were residents of Sichuan Province. For the evaluation of the treatment effects of an oral calcium supplement in children with high blood lead and mild lead poisoning, 73 patients (100 ≤ BLLs ≤ 249 μg/L) received subsequent check-ups.

### 2.3. Animals

BALB/c mice were selected as experimental subjects. Live cell imaging was performed to assess the dynamics of Ca^2+^ signals inside neurons in the cerebral cortex. Deionized drinking water and food were freely accessible to the mice throughout the study. The mice were housed at a constant temperature (22 ± 2 °C) and relative humidity (50% ± 10%) with a 12-h light/dark cycle.

### 2.4. BLL Test

All tests were performed in the Department of Laboratory Medicine, West China Second University Hospital of Sichuan University, China. This laboratory subscribes to the National System of External Quality Assessment and the College of American Pathology. Whole blood samples were drawn from the children into lead-free vacutainer tubes that contained sodium heparin. The BLLs were analyzed using atomic absorption spectrometry (BH2100, Bohui Co., Ltd., Beijing, China) with the matched calibrator and quality control reagent provided by the manufacturer. Quality control reagent was tested before, during, and after sample testing to ensure the accuracy of the results. We participate in the National Health Commission’s external quality assessment of BLL tests every year. Only laboratories that pass the assessment can continue their tests.

### 2.5. Follow-Up

Currently, children with BLLs of 100 μg/L or higher need to be treated in China. Treatment is divided into two categories. The first is chelation therapy for children with BLLs greater than or equal to 250 μg/L. The second is oral calcium supplement for children with BLLs less than 250 μg/L. Because chelation therapy has been proven to be a rapid and effective treatment, only oral calcium supplementation was evaluated in this study. In order to evaluate the treatment effects in children with high blood lead and mild lead poisoning, 73 patients (100 ≤ BLL ≤ 249 μg/L) aged 2–7 years received subsequent check-ups, among whom 54 had 100 ≤ BLL ≤ 199 μg/L, and 19 had 200 ≤ BLL ≤ 249 μg/L. Children with 100 ≤ BLL ≤ 199 μg/L were given 330 mg calcium per day, and children with 200 ≤ BLL ≤ 249 μg/L were given 440 mg calcium per day. After a period of treatment, BLLs of the children were tested again in our hospital. Treatment was considered effective if BLL was lower than 100 μg/L and ineffective if still greater than or equal to 100 μg/L.

### 2.6. Ca^2+^ Imaging

Ca^2+^ imaging is a powerful tool to measure neuronal dynamics and network activity [13]. First, artificial cerebrospinal fluid (aCSF) and Ca^2+^ stain were prepared. The aCSF comprised 7.25 g NaCl, 0.335 g KCl, 1.8 g glucose, 0.144 g NaH_2_PO_4_, 2.18 g NaHCO_3_, 0.222 g CaCl_2_, and 0.095 g MgCl_2_ (Shanghai Aladdin Biochemical Technology Co., Ltd., Shanghai, China) in 1 L distilled water. The Ca^2+^ stain comprised 15 μL Pluronic F-127 (100 mg in 0.5 mL dimethylsulfoxide (DMSO)) (Shanghai Aladdin Biochemical Technology Co., Ltd., Shanghai, China), 15 μL Fluo-4 AM (1.4 μL in 13.6 μL DMSO) (Biyuntian Biotechnology Co., Ltd., Shanghai, China), and 3 mL aCSF. Second, solutions with 100 and 50 μg/L concentrations of lead were prepared by dissolution of lead acetate with aCSF, producing 100 and 50 μg/L lead solutions of 500 mL.

Mice were euthanized on post-natal day 21 via cervical dislocation, and their whole brains were isolated and harvested. Coronal slices of somatomotor areas in the brain were cut at a thickness of 100 μm in aCSF, which maintained a temperature of 37 °C and bubbled with 95% O_2_/5% CO_2_. Brain slices were incubated in the Ca^2+^ stain for 30 min at 37 °C, and they were then washed three times with aCSF for 10 min. Finally, the coronal slices were observed using a positive fluorescence microscope (A1R + MP, Eclipse 80i, Nikon Corp., Tokyo, Japan). The result was recorded by taking pictures every 5 s. Fifteen subjects were equally divided into three groups according to the concentrations of lead solutions: 100 μg/L, 50 μg/L, and control groups (only aCSF). The coronal slices were stabilized for 2 min in aCSF, then aCSF was replaced with lead solutions in the 100 μg/L group and 50 μg/L group. After the coronal slice was soaked in the lead solution for 30 s, the lead solution was switched with aCSF. The experiment ended after 15 min of observation in aCSF. The control group was observed only in aCSF. Ca^2+^ imaging was repeated three times.

### 2.7. Statistical Analysis

The median and interquartile range (IQR) were used to describe the BLL in children, which was not normally distributed. The Kruskal–Wallis test was used to compare the BLLs of different age groups, and the Mann–Whitney U test was used to compare the data of two groups. The chi-square was used to compare the boy to girl ratios between different years. The treatment efficacy of oral calcium supplements in children with high BLLs was analyzed using the Kaplan–Meier method. All analyses were performed using SPSS 19.0 (IBM, Inc., New York, NY, USA). All statistical tests were two-sided, and a *p*-value less than 0.05 was considered statistically significant.

## 3. Results

### 3.1. Median BLL

**Figure 1:**
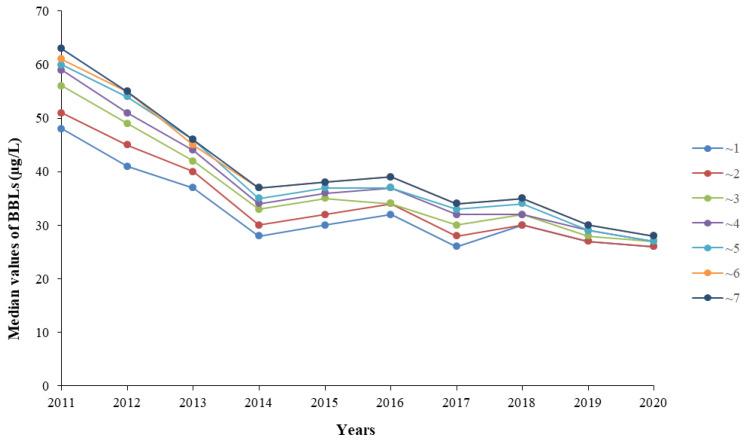
Changes in the median BLL of different age groups from 2011 to 2020. The seven colors represent seven age groups. Blue represents ~1 year group; Red represents ~2 years group; Green represents ~3 years group; Purple represents ~4 years group; Light blue represents ~5 years group; Orange represents ~6 years group; Navy blue represents ~7 years group.

A total of 76,362 children, among whom 43,993 (57.6%) were boys and 32,369 (42.4%) were girls, were included in the study (Table 1). The overall boy to girl ratio was 1.36. The boy to girl ratio in each year were as follows: 1.36 in 2011, 1.37 in 2012, 1.32 in 2013, 1.34 in 2014, 1.30 in 2015, 1.38 in 2016, 1.37 in 2017, 1.31 in 2018, 1.48 in 2019, and 1.41 in 2020. The boy to girl ratios were not significantly different from 2011 to 2018 (χ^2^ = 6.82, *p* = 0.448). The boy to girl ratio in 2019 was significantly higher than those in 2011–2018 (χ^2^ = 16.41, *p* = 0.037). The average age was 3.3 ± 1.8 years old. The distribution of BLL was positively skewed. The median BLL was 35 μg/L (IQR: 28–47), and the range was 8–963 μg/L. Table 1 represents the BLLs of boys and girls in different years; the median BLL of boys was significantly higher than that of girls (*p* < 0.05). The median BLLs for different age groups are illustrated in Table 2. The median BLL was lowest (32 μg/L) in the ~1 year group and highest (38 μg/L) in the ~6 and ~7 years groups (*p* < 0.001). The median BLLs were not significantly different between the ~6 and ~7 years groups (Z = −0.22, *p* = 0.826). Overall, the median BLLs increased with age in all children. Figure 1 shows the changes in the median BLL of different age groups from 2011 to 2020. The median BLLs in each year are as follows: 55 μg/L (IQR: 44–67) in 2011, 47 μg/L (IQR: 37–60) in 2012, 41 μg/L (IQR: 33–52) in 2013, 33 μg/L (IQR: 27–39) in 2014, 34 μg/L (IQR: 29–41) in 2015, 35 μg/L (IQR: 30–44) in 2016, 30 μg/L (IQR: 26–36) in 2017, 32 μg/L (IQR: 26–40) in 2018, 29 μg/L (IQR: 25–35) in 2019, and 27 μg/L (IQR: 24–31) in 2020.

### 3.2. Elevated BLLs

**Figure 2:**
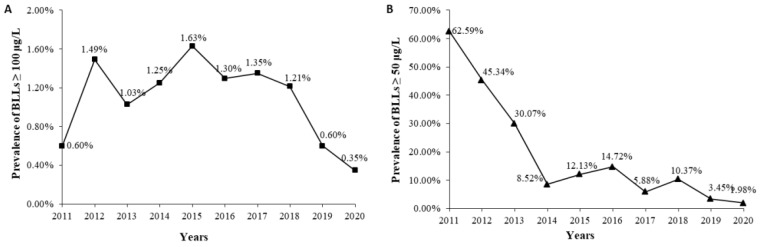
Prevalence of BLLs ≥ 100 μg/L (**A**) and BLLs ≥ 50 μg/L (**B**) from 2011 to 2020.

Because the standards are different between the Ministry of Health of China and the CDC of the United States, we analyzed the prevalence of two BLLs. The overall prevalence of BLLs ≥ 100 μg/L was 1.20%. The prevalence of BLLs ≥ 100 μg/L varied from 1.63% to 0.35% during the last decade. It was the lowest in 2020 and highest in 2015. There was no trend in prevalence from 2011 to 2015, but it has been decreasing year by year since 2015 (Figure 2A). The overall prevalence of BLLs ≥ 50 μg/L was 21.56%. The prevalence of BLLs ≥ 50 μg/L varied from 62.59% to 1.98% during the last decade, and it was highest in 2011 and lowest in 2020. There was a downward trend of the prevalence in general, and it presented a sharp fall from 2011 to 2014 (Figure 2B). The trend of change in the prevalence of BLLs ≥ 50 μg/L is consistent with that in the median BLLs.

### 3.3. Treatment Efficiency

**Figure 3:**
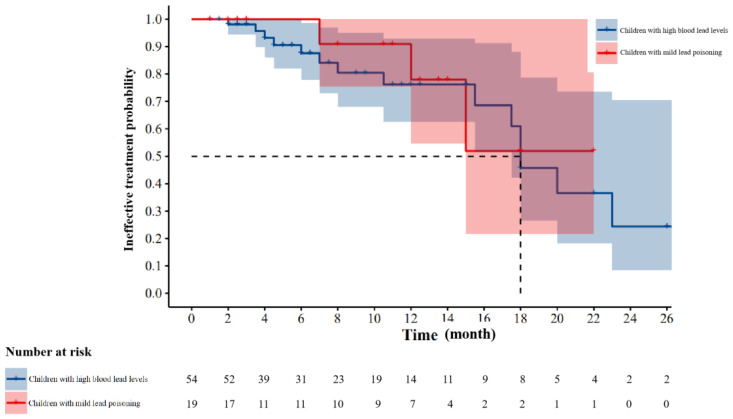
Treatment efficiency of oral calcium supplements in children with high BLLs and mild lead poisoning.

To evaluate treatment efficiency, 73 participants were recruited into two groups. The first included 54 children with high blood lead (100 ≤ BLLs ≤ 199 μg/L), and the second included 19 children with mild lead poisoning (200 ≤ BLLs ≤ 249 μg/L). After a period of oral calcium supplement, the BLLs of the 73 children were retested, which was the basis for us to evaluate treatment efficiency. During the follow-up, 15 children had their BLLs reduced to blow 100 μg/L in the first group, and only three children had their BLLs reduced to blow 100 μg/L in the second group. The shortest time required for an effective treatment in the first and second groups was two and seven months, respectively. The interval from the beginning of treatment to the retesting of BLLs varied in each child, ranging from 1 month to 26 months with a mean of 8.6 months. The result exhibited no statistical difference between the two groups (χ^2^ = 0.273, *p* = 0.601). The median time required for an effective treatment was 18 months in the first group. The median time required for an effective treatment was not available in the second group (Figure 3).

### 3.4. Lead Exposure Disrupted Ca^2+^ Signals in Developing Cortical Neurons

**Figure 4:**
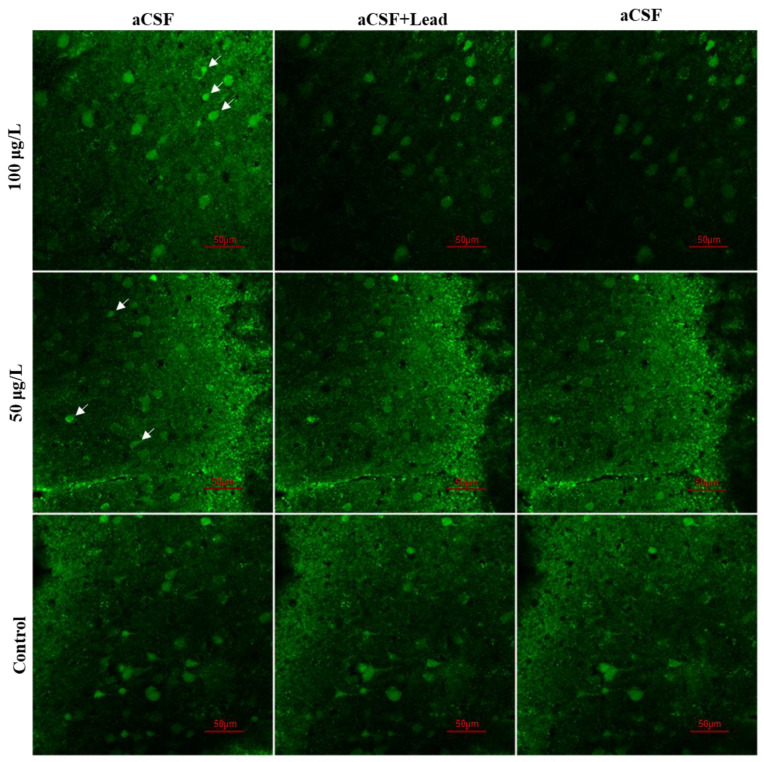
Representative confocal images of Ca^2+^ in the brain slices. The cells indicated by the white arrows are neurons with reduced Ca^2+^ signaling after lead exposure. Dynamic loss of Ca^2+^ fluorescence intensity can be observed from left to right: the left column shows the fluorescence intensity of brain slices before lead exposure, the middle column shows the fluorescence intensity after lead exposure, and the right column shows the fluorescence intensity after the removal of lead exposure. The control group was observed in aCSF. Scale bar, 50 μm.

A mechanistic study was performed on mice to further clarify the significant neurotoxicity of lead. Lead not only competes for calcium binding sites but also reduces the level of intracellular calcium [14], which prompted us to measure the acute effects of lead exposure on developing cortical neurons. The Ca^2+^ stain was used in coronal slices of mice on post-natal day 21. An assessment of the acute effects of lead on neuron Ca^2+^ signals was achieved via the continuous infusion of lead. Interestingly, we observed that lead exposure led to a gradual and persistent loss of Ca^2+^ signals in developing cortical neurons. All fluorescent patterns of the excited neurons indicated by the white arrows disappeared. The fluorescent intensity in the 100 μg/L group was lower than that of the 50 μg/L group and control group after lead exposure, and it did not recover even after the aCSF (including CaCl_2_) was switched back (i.e., the lead exposure was removed) (Figure 4). The change in fluorescence intensity in the 50 μg/L group was not observable. The fluorescent patterns of only a few of the excited neurons indicated by the white arrows disappeared.

## 4. Discussion

Lead has become known as a highly toxic element for children, as it has no known biological function. Child intoxication occurs through the air, water, and skin by inhaling polluted air or eating contaminated food. The clinical features of lead intoxication are non-specific and often go unrecognized [15]. We analyzed BLL data for 76,362 children, and the overall median BLL was 35 μg/L from 2011 to 2020. A downward trend in basic BLLs was observed. The BLLs of boys were higher than that of girls every year, which is consistent with the findings of a previous study [16]. The boy to girl ratio was the highest in 2019, but the median BLL was the second lowest. It suggested that the sex ratio is not the decisive factor in determining the median BLL. The BLLs increased with age, which suggests that lead accumulates in the body and has cumulative effects [17]. Sichuan Province is an area with low prevalence of BLL ≥ 100 μg/L; therefore, BLLs in children are significantly lower compared with the other parts of China. For example, the mean BLL was 44.75 μg/L in Wuhan among 15,536 children [18]. However, lead-poisoning prevention is still an urgent task in Sichuan Province when compared with developed countries [19,20].

Even at relatively low blood concentrations, shown significant associations have been shown between lead and such adverse effects as intellectual impairment, hyperactivity and lack of concentration, and damage to virtually all organ systems, including brain, heart, kidneys, liver, and the circulatory system. Elevated blood lead concentrations have also been associated with lowered birth weight, violent or aggressive behavior, hypertension, and so on [21]. The prevalence of children with BLL ≥ 100 μg/L was low [22], and this value did not decrease annually. Notably, the prevalence of children with BLL ≥ 100 μg/L was 0.6% in 2011; however, the BLL value was at its highest. This manifests two things: first, the basic BLLs of children in 2011 were relatively high, even though the prevalence of children with BLL ≥ 100 μg/L was low; second, children primarily exhibited BLL ≥ 100 μg/L owing to accidental lead exposure, which has little influence on the overall level of the whole population. The prevalence of children with BLL ≥ 50 μg/L was the highest in 2011 and lowest in 2020. It has gradually decreased in the past decade. The trend of change in the prevalence of BLLs ≥ 50 μg/L is consistent with that in the median BLLs. The prevalence of BLLs ≥ 50 μg/L indicates the change of median BBLs more accurately than the prevalence of BLLs ≥ 100 μg/L. Our results accurately represent the BLLs of children in Sichuan Province. With the improvement of living standards, people pay increasing attention to lead poisoning; thus, the BLLs of children and the prevalence of children with BLL ≥ 50 μg/L are well controlled.

The BLLs of children have gradually decreased in the past decade. In regards to the previously mentioned guidelines issued by the Ministry of Health, China, since the prevention, diagnosis, and treatment of lead poisoning was based on the situation in China in 2006, the content of articles may require updating. Moreover, the documents did not include the possibility of standardized nutritional interventions for children with high blood lead and mild lead poisoning. Meanwhile, this study explored the utility of calcium supplements in the treatment of lead poisoning. The median time required for the effective treatment of children with high blood lead using a calcium supplement was 18 months. The median time for the effective treatment of children with mild lead poisoning was not available owing to the small number of subjects and short follow-ups. The shortest time required for an effective treatment in children with mild lead poisoning was seven months, which was longer than two months. It was assumed that the median time required for the effective treatment of children with mild lead poisoning must be longer than that of children with high blood lead. The result exhibited no statistical difference between the two groups. This is obviously unreasonable and the result may be influenced by the small number of subjects or short follow-ups. Lead can damage the nervous system in the development period, and long-term exposure is bound to cause immeasurable effects on the nervous systems of children. The duration of treatment should be as short as possible. Chelation therapy has been proven to be a rapid and effective treatment. Maybe we can adjust the BLL for chelation therapy appropriately; for example, chelation therapy was adopted when the BLL ≥ 150 μg/L. We intended for the results of this study to highlight the need for a new standard treatment that will shorten the treatment time as much as possible.

Lead is regarded as an important environmental risk factor leading to irreversible neurological disorders, such as Alzheimer’s disease, Parkinson’s disease, amyotrophic lateral sclerosis, attention deficit hyperactivity disorder, etc. [23]. It can cause a variety of adverse outcome on central nervous system (CNS). Lead mimics the function of Ca^2+^ when it enters the body, which results in a range of specific documented long-term deleterious effects [24], such as cognitive decline, hypertension, and cardiovascular disease [17]. For children, the most serious effect caused by lead is neurological damage. Using Ca^2+^ imaging experiments, we have dynamically observed that acute lead exposure leads to a rapid and irreversible decrease in the concentration of Ca^2+^ in neurons in the cerebral cortex. The potential mechanism that causes this is as follows: lead binds to the selectivity filter of the voltage-gated calcium channels, and when the binding affinity of lead for the binding site is high, some Pb^2+^ may enter the cell through these channels. Elevated intracellular lead levels result in the substitution of Ca^2+^, and it blocks the voltage-gated calcium channels [24]. Thus, after the lead exposure was removed, and CaCl_2_ was added as a treatment, the neurons did not recover within a short period of time. Although only a limited amount of evidence directly suggested loss of Ca^2+^ in lead-induced neurological disorders, loss of Ca^2+^ may still constitute important aspects connecting environmental lead exposure and CNS-related disease.

## 5. Conclusions

This study focused on the current BLLs of children in Sichuan Province and the antagonistic effects between lead and calcium regarding treatment and neurotoxicity. The BLLs of children in Sichuan Province have clearly decreased recently, and only 0.35% of children have BLLs higher than 100 μg/L in 2020. Sichuan Province is now a low-epidemic area in terms of lead pollution. The oral calcium supplement treatment given to children with high BLLs and mild lead poisoning takes a long time to remove lead. Therefore, a new strategy is urgently needed to shorten the treatment time. We also demonstrated the neurotoxicity of lead using Ca^2+^ imaging, which dynamically displayed a rapid and irreversible decrease in the concentration of Ca^2+^ in neurons. We propose that these results can provide support for local illness prevention and policy making. Our study has some limitations. First, as all subjects were outpatients for regular checkups in our hospital, there was a selection bias in this study. Second, the median time for effective treatment of children with mild lead poisoning was not available owing to the small number of subjects and short follow-ups. In future research, we will expand the follow-up population and extend the duration of follow-up to obtain a more accurate median time for effective treatment. Further basic experiments will be carried out to further reveal the neurotoxic mechanism of lead by means of electrophysiological experiments, neurotransmitter content detection, and gene sequencing.

## Figures and Tables

**Table 1:** Comparison of BLLs between boys and girls from 2011 to 2020.

Years	Boy		Girl		*p*-Value
	N	Median (P25–P75)	N	Median (P25–P75)	
2011	4797	56 (45–68)	3525	53 (43–65)	<0.001
2012	5818	49 (38–61)	4236	46 (36–58)	<0.001
2013	4818	42 (34–54)	3655	40 (32–51)	<0.001
2014	4899	33 (27–39)	3661	32 (27–38)	<0.001
2015	5031	35 (29–42)	3861	34 (28–40)	<0.001
2016	4286	36 (30–45)	3114	35 (29–42)	<0.001
2017	4106	31 (26–36)	3000	30 (26–36)	<0.001
2018	2800	33 (26–40)	2139	32 (26–40)	0.01
2019	2890	29 (25–35)	1957	28 (25–34)	0.009
2020	4548	27 (24–32)	3221	26 (23–31)	<0.001
Total	43,993	36 (28–48)	32,369	35 (28–46)	<0.001

**Table 2:** The BLLs (μg/L) of children in different age groups during 2011–2020.

Years	~1		~2		~3		~4		~5		~6		~7		*p*-Value
	N	Median (P25–P75)	N	Median (P25–P75)	N	Median (P25–P75)	N	Median (P25–P75)	N	Median (P25–P75)	N	Median (P25–P75)	N	Median (P25–P75)	
2011	1575	48 (38–57)	1726	51 (42–62)	2220	56 (45–67)	901	59 (48–70)	663	60 (48–73)	658	61 (50–73)	579	63 (52–76)	<0.001
2012	2210	41 (33–51)	2265	45 (36–56)	2323	49 (38–61)	1065	51 (41–64)	835	54 (43–66)	710	55 (44–68)	646	55 (43–68)	<0.001
2013	1834	37 (30–46)	2051	40 (31–50)	1940	42 (34–53)	801	44 (35–57)	686	46 (38–57)	632	45 (38–57)	529	46 (39–58)	<0.001
2014	1572	28 (24–34)	1983	30 (25–37)	2173	33 (28–39)	786	34 (30–40)	710	35 (31–41)	732	37 (33–42)	604	37 (33–42)	<0.001
2015	1797	30 (25–37)	1918	32 (27–39)	2318	35 (29–42)	884	36 (32–44)	741	37 (33–44)	576	38 (33–46)	658	38 (35–47)	<0.001
2016	1275	32 (26–39)	1652	34 (28–42)	2002	34 (29–43)	850	37 (31–44)	588	37 (33–47)	525	39 (34–48)	508	39 (34–48)	<0.001
2017	1311	26 (23–32)	1220	28 (25–34)	1767	30 (26–36)	811	32 (28–37)	729	33 (29–38)	673	34 (30–39)	595	34 (30–38)	<0.001
2018	740	30 (24–37)	924	30 (25–39)	1260	32 (26–39)	606	32 (27–41)	534	34 (28–42)	464	35 (29–43)	411	35 (29–43)	<0.001
2019	592	27 (23–32)	689	27 (24–33)	1131	28 (25–34)	593	29 (25–36)	600	29 (26–35)	600	30 (27–36)	642	30 (27–36)	<0.001
2020	1176	26 (23–30)	1119	26 (23–30)	1608	27 (24–31)	915	27 (24–32)	980	27 (24–31)	989	28 (25–32)	982	28 (24–33)	<0.001
Total	14,082	32 (26–43)	15,547	34 (27–46)	18,742	36 (28–48)	8212	37 (30–50)	7066	37 (30–49)	6559	38 (30–50)	6154	37 (31–50)	<0.001

## Data Availability

All relevant data from this study are available from the authors.
